# Supplementary material for: The Effect of Meditation, Mindfulness, and Yoga in Patients with Rheumatoid Arthritis
Source: J Pers Med. 2022 Nov 15;12(11):1905. doi: 10.3390/jpm12111905 (PMC9696072; doi:10.3390/jpm12111905)
Supplement: Supplementary file 1 [file jpm-12-01905-s001.zip › jpm-1984257-supplementary.pdf]

SUPPLEMENTARY MATERIALS

File A: Search strings per database

PUBMED (including MEDLINE)
("Mind-Body Therapies"[Mesh:NoExp] OR "Meditation"[Mesh] OR "Mind Body Therap*"[tiab] OR Meditation[tiab] OR "Yoga"[Mesh] OR "yoga"[tiab] OR "Relaxation Therapy"[Mesh] OR relaxation*[tiab] OR mindfulness[tiab] OR "Mindfulness"[Mesh] OR "Relaxation"[Mesh:NoExp] OR FAM[tiab] OR OMM[tiab] OR pranayama[tiab] OR hathayoga[tiab] OR (yogic[tiab] AND sleep[tiab])) AND ("Arthritis, Rheumatoid"[Mesh:NoExp] OR "Rheumatoid Arthritis"[tiab] OR “rheumatic arthritis”[tiab] OR “chronic articular rheumatism”[tiab])
EMBASE (Embase.com)
('meditation'/exp OR 'meditation':ti,ab,kw OR 'mindfulness'/exp OR 'mindfulness':ti,ab,kw OR 'yoga'/exp OR 'yoga':ti,ab,kw OR 'relaxation training'/exp OR 'relaxation':ti,ab,kw OR 'transcendental meditation'/exp OR ‘FAM’:ti,ab,kw OR ‘OMM’:ti,ab,kw OR ‘pranayama’:ti,ab,kw OR ‘hathayoga’:ti,ab,kw OR ‘yogic sleep’:ti,ab,kw OR ‘mind body therap*’:ti,ab,kw) AND ('rheumatoid arthritis'/de OR 'rheumatoid arthritis':ti,ab,kw OR ‘rheumatic arthritis’:ti,ab,kw OR ‘chronic articular rheumatism’:ti,ab,kw)
WoS CC, Chinese science citation database & KCI-Korean Journal Database
TS =((“meditation” OR “mindfulness” OR “yoga” OR “relaxation” OR “FAM” OR “OMM” OR “pranayama” OR “hathayoga” OR “yogic sleep” OR “mind body therap*”) AND (“rheumatoid arthritis” OR “rheumatic arthritis” OR “chronic articular rheumatism”))
CINAHL (EBSCO)
((MH "Meditation") OR (MH "Yoga+") OR (MH "Relaxation Techniques") OR (MH "Mind Body Techniques") OR (MH "Mindfulness") OR TI(“meditation” OR “mindfulness” OR ”yoga” OR “relaxation” OR “FAM” OR “OMM” OR “pranayama” OR “hathayoga” OR “yogic sleep” OR “mind body therap*”) OR AB(“meditation” OR “mindfulness” OR ”yoga” OR “relaxation” OR “FAM” OR “OMM” OR “pranayama” OR “hathayoga” OR “yogic sleep” OR “mind body therap*”)) AND ((MH "Arthritis, Rheumatoid") OR TI(“rheumatoid arthritis” OR “rheumatic arthritis” OR “chronic articular rheumatism”) OR AB(“rheumatoid arthritis” OR “rheumatic arthritis” OR “chronic articular rheumatism”))

File B: Quality assessment tools

The PEDro scale consists of 11 items which are evaluated with ‘yes’, or one point, versus ‘no’, or zero points, giving a maximal global score of 11.

The MINORS tool uses a scale from 0 to 2: a score of 0 is given to criteria that are not reported; a score of 1 is given to inadequately reported criteria; a score of 2 is given to adequately reported criteria. This gives a maximum score of 16 and 24, for noncomparative and comparative studies, respectively.

The JBI tool for cross-sectional studies does not include a numeric scale; therefore, we adapted the following scoring system. There are 8 criteria with 4 rating options per criterium: ‘yes’ when the quality criterium was fulfilled; ‘no’ when the quality criterium was not met; ‘unclear’ when the criterium was not straightforward; ‘not applicable’ when the

criterium did not apply to the specific study. A score of 1 point was given to the 'yes' rating option and a score of 0 points was given to the other three rating options (no, unclear, not applicable), giving a maximum score of 8 points.

File C: Scores per study per individual question per tool

**PEDro Scale**	Eligibility criteria	Random allocation	Concealed allocation	Similar groups (baseline)	Blind subjects	Blind therapists	Blind assessors	<15% loss to follow-up	Intention-to-treat	Between-group comparisons	Point and variability measures
Puksic, et al. (2021)	Green	Green	Green	Green	Red	Red	Green	Green	Green	Green	Green
Davis, et al. (2015)	Green	Green	Green	Green	Red	Red	Red	Green	Red	Green	Green
Ward, et al. (2018)	Green	Green	Green	Green	Red	Red	Green	Green	Red	Green	Green
Dalili, et al. (2019)	Green	Green	Red	Red	Red	Red	Red	Green	Green	Green	Green
Gautam, et al. (2021)	Green	Green	Green	Green	Red	Red	Green	Green	Green	Green	Green
Barsky, et al. (2010)	Green	Green	Green	Green	Red	Red	Green	Red	Green	Green	Green
Singh, et al. (2011)	Green	Green	Red	Green	Red	Red	Red	Green	Red	Green	Green
Fogarty, et al. (2015)	Green	Green	Red	Green	Red	Red	Green	Red	Red	Green	Green
Ganesan, et al. (2020)	Green	Green	Green	Green	Red	Red	Red	Green	Red	Green	Green
Haslock, et al. (1994)	Red	Red	Red	Green	Red	Red	Green	Green	Green	Green	Green
Evans, et al. (2013)	Green	Green	Green	Red	Red	Red	Green	Red	Red	Green	Green
Fogarty, et al. (2019)	Green	Green	Red	Green	Red	Red	Green	Red	Green	Green	Green
Gautam, et al. (2020)	Green	Green	Green	Green	Red	Red	Green	Green	Green	Green	Green
Pradhan, et al. (2007)	Green	Green	Green	Red	Red	Red	Green	Green	Green	Green	Green

Red = No (0 points); green = yes (1 point).

**MINORS tool**	Clear aim	Inclusion consecutive patients	Prospective collection	Appropriate endpoints	Unbiased assessment	Appropriate follow-up period	<5% loss to follow-up	Prospective sample calculation	Adequate control group	Contemporary groups	Similar groups (baseline)	Adequate statistics
Bosch, et al. (2009)	Green	Green	Green	Orange	Red	Orange	Orange	Red	Green	Green	Orange	Green
Telles, et al. (2011)	Green	Green	Green	Green	Red	Orange	Green	Red				
Yazdani, et al. (2017)	Green	Green	Green	Green	Red	Green	Red	Orange	Green	Green	Green	Green
Zautra, et al. (2008)	Green	Green	Green	Green	Green	Green	Orange	Red	Green	Green	Orange	Green
Bagheri-Nesami, et al. (2006)	Green	Green	Green	Green	Red	Orange	Orange	Green	Green	Green	Green	Red
Evans, et al. (2010)	Green	Green	Green	Green	Red	Orange	Orange	Red				
Badsha, et al. (2009)	Green	Green	Green	Green	Orange	Orange	Green	Red	Green	Green	Green	Orange
Dash, et al. (2001)	Green	Red	Red	Green	Red	Green	Red	Red	Green	Orange	Orange	Red

Red = not reported (0 points); orange = reported but inadequate (1 point); green = reported and adequate (2 points). The last four columns are additional criteria in the case of comparative studies.

**JBI**	Eligibility criteria	Details subjects and setting	Valid exposure	Standard criteria for conditions	identification confounders	Approach confounders	Valid outcome	Adequate statistics
Puksic, et al. (2021)	Green	Green	Red	Green	Green	Green	Red	Green

Red = No (0 points); green = yes (1 point).

Supplementary file D: main conclusion and limitations per included study

Author (year)	Main conclusion(s)	Limitation(s)
	Yoga	
Haslock (1994)	Non-significant improvement in hand grip strength for RA patients practicing yoga	• Letter-to-editor<br>• Relatively old paper<br>• Limited sample size<br>• No control group
Dash (2001)	Improvement in hand grip strength for RA patients practicing yoga	• Intervention group had lower hand grip strength than controls<br>• Limited sample size<br>• Very intensive yoga approach<br>• Relatively young RA patients
Badsha (2009)	Patients had statistically significant improvements in DAS28 and HAQ, but not QOL	• Pilot study<br>• expectation bias due to non-blinding of patients/rheumatologists<br>• Limited sample size
Bosch (2009)	A decreased HAQ disability index, decreased perception of pain and depression, and improved balance. No change in awakening or diurnal cortisol patterns	• Pilot study<br>• Limited sample size<br>• Only women<br>• Motivated yoga participants
Evans (2010)	Improvements in pain, pain disability, depression, mental health, vitality, and self-efficacy	• High attrition<br>• Very low sample size<br>• No control group<br>• Relatively young RA patients
Singh (2011)	Improvements in pain and selected blood parameters, not in joint counts or CRP	• Limited information supplied in article, questionable peer-review.<br>• Relatively young RA patients<br>• Yogic package included more than just yoga
Telles. (2011)	Reduced disability scores of the HAQ and rheumatoid factor levels, with an increase in bilateral hand grip strength in male participants alone. No improvement of CRP level	• No control group<br>• Motivated yoga participants
Evans. (2013)	Improvement on standardized measures of HRQoL, pain disability, general health, mood, fatigue, acceptance of chronic pain, and self-efficacy regarding pain	• Limited sample size<br>• Only women<br>• Yoga group had experienced RA for longer and was more disabled than the control group
Ward (2018)	No effect of yoga compared with usual care in physical and psychosocial outcomes	• Limited sample size<br>• Selected patients with sleep and pain problems
Greysen (2019)	More likely to work full-time, less likely to be unable to work due to disability, and better physical function	• interviewer administered self-assessment patient-reported outcome (PRO) measures by phone

		• Participants derived from private rheumatology clinic;primarily white with relatively high income and education
Gautam (2020)	Reduction in DAS28-ESR; improvement seen in the physical health, psychological, social relationships domains of QOL, except environmental; downregulation of IL-6, TNF-α, and CTLA4 and upregulation of TGF-β.	• Intensive yoga exercises<br>• No blinding
Ganesan (2020)	Decreased DAS28ESR IL-1α and cortisol decreased, better sympathovagal balance	• Only stable patients<br>• Relatively early RA (<3y)
Gautam (2021)	Improvement in DAS28ESR, HAQ and depression	• Only active RA (DAS>2.6)
Puksic (2021)	Improvement in fatigue depression and anxiety<br>No improvement in SF-36 or pain	• Active control group received less time per session<br>• Limited sample size
	Mindfulness	
Pradhan (2007)	No difference in depressive symptoms, psychological distress, well-being, DAS28, and mindfulness.	• Recruitment not via rheumatology clinic<br>• Limited sample size
Zautra (2008)	Improvement in coping efficacy;<br>RA patients with recurrent depression benefited most	• Participants with low-income<br>• Mix of cognitive behavioural therapy and mindfulness blurs effect
Davis (2015)	Improvements in daily pain and stress reactivity	• Mix of cognitive behavioural therapy and mindfulness blurs effect
Fogarty (2015)	Reduced disease activity parameters including tender joints, patient global assessment, stiffness, pain but not swollen joints, CRP	• Letter-to-editor<br>• Limited sample size
Dalili (2019)	Improved Illness Perception, Depression, Anxiety and Stress	• Limited sample size<br>• Selection bias as Iranian participants had to have at least a diploma
Fogarty (2019)	Improved DAS28 mediated by depression, not anxiety	• Limited sample size<br>• Low levels of anxiety and depression
	Relaxation	
Bagheri-Nesami (2006)	Improved anxiety, depression and feeling of well-being. Changes in clinical symptoms and laboratory findings	• Established RA (>2y)
Barsky (2010)	No difference from cognitive-behaviour therapy or arthritis education.<br>Pain improved	• Participants receiving psychosocial treatment for RA were excluded
Yazdani (2017)	All SF-36 scores improved but bodily pain and physical constraint	• Active group showed worse scores for mental health at baseline
